# Complete Genome Sequence of Deinococcus aquaticus Type Strain PB314, a Non-Extremophile Representative of the Genus *Deinococcus*

**DOI:** 10.1128/mra.00026-23

**Published:** 2023-06-08

**Authors:** Chad Albert, Jonathan Hill, Leilani Boren, Stacy Scholz-Ng, Nahid Fatema, Ryan Grosso, Erica Soboslay, James Tuohy

**Affiliations:** a Biology Department, Glendale Community College, Glendale, Arizona, USA; University of Arizona

## Abstract

Here, we present the complete genome of Deinococcus aquaticus PB314^T^, an aquatic mesophilic bacterium, assembled using Oxford Nanopore Technologies (ONT) long-read and Illumina short-read sequencing platforms. The hybrid assembly predicts 3,658 genes, located across 5 replicons with an overall G+C content of 68.82%.

## ANNOUNCEMENT

The genus *Deinococcus* (1) currently contains over 89 species based upon validly published names (https://www.bacterio.net/genus/deinococcus). Deinococcus aquaticus is one of only a few species in this genus not known to be either a polyextremophile or polytolerant (2, 3). Therefore, the description of the complete genome of this species will serve as a high quality reference against which to compare the genomic organization of related deinococci that exhibit extremophile phenotypes (2).

*D. aquaticus* PB314^T^ is a gram-negative, rod-shaped, non-spore-forming bacterium first isolated from a freshwater stream of the River Gapcheon, South Korea (4). A freeze-dried sample of *D. aquaticus* (DSM 18376) was obtained from the German Collection of Microorganisms and Cell Cultures (DSMZ) and grown in tryptone-glucose-yeast extract (TGY) medium for 24 h at 30°C. Cells were pelleted, resuspended, and grown in fresh TGY until an *A*_600_ value of 0.8 ± 0.2 was achieved. Cells were lysed using a Bead Mill 4 bead-beater (Fisher Scientific, USA) for 60 s at a rate of 5 m/s. DNA extraction was completed using the DNeasy Ultraclean microbial kit (Qiagen, Germany). DNA concentration and quality were assessed using a Qubit 4.0 fluorimeter with a Qubit dsDNA BR kit (Thermo Fisher, USA) as well as with a NanoDrop ONE (Thermo Fisher, USA).

Nanopore sequencing (Oxford Nanopore Technologies [ONT]) was performed using a Rapid Sequencing Kit (SQK-RAD004) for library preparation. The sample was loaded onto an R10.3 FLO-MIN111 flow cell on a MinION Mk1C sequencing platform. For sequence planning and data acquisition, MinKNOW v21.11.6 (ONT) was used, with fast base-calling mode enabled. All other settings remained at default. Illumina sequencing was performed on the MiSeq platform on a 2- by 250-bp flow cell using the Hyperplus KK8514 preparation kit (Kapa Biosystems).

The Galaxy bioinformatics suite (https://usegalaxy.org.au) provided raw sequence processing and annotation (5–8). Processed Nanopore and Illumina data were piped into Unicycler v0.5.0+galaxy1 for hybrid *de novo* sequence assembly (5, 9). Prokka v1.14.6+galaxy1 (5, 10) and NCBI’s Prokaryotic Genome Annotation Pipeline (PGAP) (11) were used for genome annotation. Prokka provided an initial rapid draft annotation while PGAP provided a comprehensive and accurate annotation of the genome. Illumina and ONT sequencing provided 10,082,283 and 344,770 reads, respectively, with Nanopore reads having an *N*_50_ of 3.37 kb.

Assembly quality was determined with QUAST v5.2.0+galaxy0 (12) and the Bacterial and Viral Bioinformatics Resource Center’s BV-BRC v3.27.7 (https://www.bv-brc.org) (13). The hybrid assembly showed high quality and contiguity with a total aligned length of 3,874,397 bp and an *N*_50_ of 3.13 Mbp, consisting of 5 replicons (Table 1), confirmed by Ori-Finder v2022 (14). Gene content was assessed by BUSCO v5.3.2+galaxy0 (15) and showed 90.3% completeness (single copy, 90.3%; duplicate, 0.0%; fragmented, 4.8%; missing, 4.9%; *n* = 124). The hybrid assembly predicted 3,658 genes (3,572 protein-coding sequences [CDS], 9 rRNA genes, 50 tRNA genes, 2 noncoding RNA [ncRNA] genes, and 25 pseudogenes), with an overall G+C content of 68.82%. Average nucleotide identity (11) analysis determined that Deinococcus seoulensis 16F1E^T^ is the closest relative of *D. aquaticus* PB314^T^ in the genus (value of 91.84%), confirming previous 16S rRNA sequence analysis (16).

**TABLE 1:** Replicon characteristics of Deinococcus aquaticus type strain PB314 (DATS)

Replicon	Length (bp)	CDS count	tRNA count	G+C content (%)	Accession no.
DATS1	3,131,215	2,947	50	68.93	CP115165
pDATS01	450,606	338	0	70.13	CP115166
pDATS02	186,976	233	0	66.23	CP115167
pDATS03	98,187	75	0	64.48	CP115168
pDATS04	7,413	4	0	63.83	CP115169

Total	3,874,397	3,597	50		

### Data availability.

The raw data, found under BioProject accession number PRJNA839771, were submitted to the NCBI Sequence Read Archive (SRA) under two experiment accession numbers: SRX18673651 (Illumina fastq files) and SRX18678867 (Nanopore reads in fastq format).
